# Antibacterial and Anti-Inflammatory Activity of an Antimicrobial Peptide Synthesized with D Amino Acids

**DOI:** 10.3390/antibiotics9120840

**Published:** 2020-11-24

**Authors:** Jlenia Brunetti, Veronica Carnicelli, Alessia Ponzi, Antonio Di Giulio, Anna Rita Lizzi, Loredana Cristiano, Laura Cresti, Giovanni Cappello, Simona Pollini, Lara Mosconi, Gian Maria Rossolini, Luisa Bracci, Chiara Falciani, Alessandro Pini

**Affiliations:** 1 Department of Medical Biotechnologies, University of Siena, 53100 Siena, Italy; cappello5@student.unisi.it (G.C.); luisa.bracci@unisi.it (L.B.); chiara.falciani@unisi.it (C.F.); alessandro.pini@unisi.it (A.P.); 2 Department of Biotechnological and Applied Clinical Sciences, University of L’Aquila, 67100 L’Aquila, Italy; veronica.carnicelli@univaq.it (V.C.); alessiapo@virgilio.it (A.P.); antonio.digiulio@univaq.it (A.D.G.); annarita.lizzi@univaq.it (A.R.L.); 3 Department of Life, Health and Environmental Sciences, University of L’Aquila, 67100 L’Aquila, Italy; loredana.cristiano@univaq.it; 4 SetLance srl, Toscana Life Sciences, 53100 Siena, Italy; cresti@setlance.com; 5 Department of Experimental and Clinical Medicine, University of Florence, 50134 Florence, Italy; simona.pollini@unifi.it (S.P.); lara.mosconi.153@gmail.com (L.M.); gianmaria.rossolini@unifi.it (G.M.R.); 6 Microbiology and Virology Unit, Careggi University Hospital, 50134 Florence, Italy; 7 Laboratory of Clinical Pathology, Santa Maria alle Scotte Hospital, 53100 Siena, Italy

**Keywords:** anti-inflammatory activity, antibacterial agents, antimicrobial peptides

## Abstract

The peptide SET-M33 is a molecule synthesized in tetra-branched form which is being developed as a new antibiotic against Gram-negative bacteria. Its isomeric form with D amino acids instead of the L version (SET-M33D) is also able to kill Gram-positive bacteria because of its higher resistance to bacterial proteases (Falciani et al., *PLoS ONE*, 2012, 7, e46259). Here we report the strong in vitro activity of SET-M33D (MIC range 0.7–6.0 µM) against multiresistant pathogens of clinical interest, including Gram-positives *Staphylococcus aureus*, *Staphylococcus saprophyticus*, and *Enterococcus faecalis*, and various Gram-negative enterobacteriaceae. SET-M33D antibacterial activity is also confirmed in vivo against a MRSA strain of *S. aureus* with doses perfectly compatible with clinical use (5 and 2.5 mg/Kg). Moreover, SET-M33D strongly neutralized lipopolysaccharide (LPS) and lipoteichoic acid (LTA), thus exerting a strong anti-inflammatory effect, reducing expression of cytokines, enzymes, and transcription factors (TNF-α, IL6, COX-2, KC, MIP-1, IP10, iNOS, NF-κB) involved in the onset and evolution of the inflammatory process. These results, along with in vitro and in vivo toxicity data and the low frequency of resistance selection reported here, make SET-M33D a strong candidate for the development of a new broad spectrum antibiotic.

## 1. Introduction

In recent years, the general misuse of traditional antibiotics has led to a rise in antimicrobial resistance. The strategies being developed to combat antimicrobial resistance include effective policies for monitoring the spread of resistance [1,2], better use of antibiotics, and intensive research and synthesis of new molecules to obtain new anti-infective therapies. Antimicrobial peptides seem to be an interesting class of molecules because they combine antimicrobial activity with low resistance selection [3,4,5]. In order to overcome the problem of peptide instability while retaining peptide properties and selectivity, researchers have changed peptide structure in various ways, such as by incorporating unnatural amino acids (e.g., D-amino acids), β-peptides or peptoids (N-substituted glycines) [6,7], or by cyclization. Several years ago, branched peptides, such as multiple antigen peptides, which have a peptidyl core of radially branched lysine residues onto which peptide sequences can be added, were synthesized [8]. These peptides have strong resistance to proteolysis which makes them particularly suitable for use in vivo [9,10,11].

The therapeutic potential of antimicrobial peptides is not limited to their antimicrobial activity, since some peptides have antitumor properties [12,13] and others are intrinsic components of innate immunity. The immunomodulatory properties of these antimicrobial peptides are also attracting much attention for the development of new anti-inflammatory drugs [14,15,16]. Inflammation is the first response of the immune system to infection or injury. Lipopolysaccharide (LPS) and lipoteichoic acid (LTA) are surface membrane components of Gram-negative and Gram-positive bacteria, respectively, which trigger the inflammatory response. LPS and LTA interact with host immune cells such as macrophages and monocytes through TLR-4 and TLR-2, producing proinflammatory cytokines, such as TNF-α, IL-1β, and IL-6 [17,18].

The synthetic peptide SET-M33L, produced with L amino acids, was previously reported to have strong antimicrobial activity in vitro and in vivo against major Gram-negative pathogens [19,20,21,22,23]. SET-M33L was synthesized in tetra-branched form for better stability in biological fluids. Its mode of action is based on a two-step mechanism: (1) high affinity binding to LPS [24]; (2) disruption of bacterial membranes [25].

**Figure 1:**
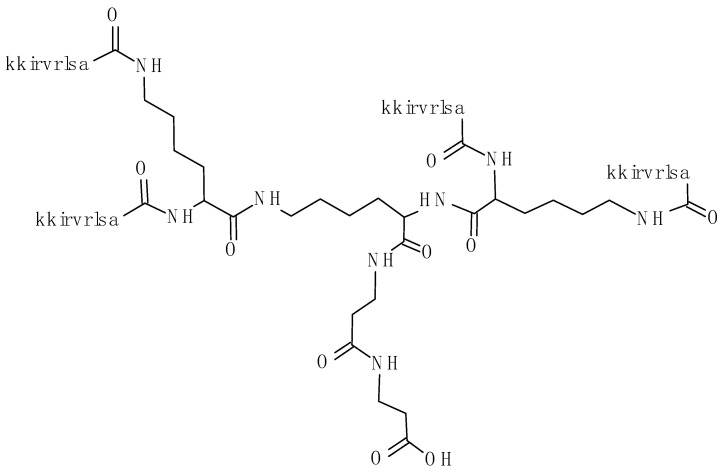
Structure of the tetra-branched SET-M33D peptide, (kkirvrlsa)_4_K_2_KβA-OH. Proportions between amino acids in the peptide sequences and the lysine core are not respected.

We also studied the tetra-branched peptide when synthesized with D amino acids (SET-M33D) (Figure 1) [26]. This peptide showed 4–16-fold higher activity than the SET-M33L against Gram-positive pathogens, including *Staphylococcus aureus* and *Staphylococcus epidermidis*, thus becoming an interesting candidate for multifunctional drug development. The increased activity shown by SET-M33D against Gram-positives compared to SET-M33L was likely due to its resistance to proteases, such as elastase, produced by Gram-positive bacteria [26].

Here we report the strong antibacterial activity of SET-M33D, and its efficacy in neutralizing LPS and LTA, thus inhibiting expression of inflammatory mediators. The resistance selection, toxicity, and efficacy in vivo of SET-M33D were also studied using an infection model with the highly virulent methicillin-resistant *S. aureus* (MRSA) strain USA 300.

## 2. Results

### 2.1. Antimicrobial Activity In Vitro

Minimum inhibitory concentrations (MICs) were determined against a collection of 41 selected isolates including reference strains and isolates of clinical origin including major Gram-positive and Gram-negative pathogens with relevant resistance genotypes (e.g., carbapenemase genes, acquired linezolid resistance gene *cfr* and novel transferable colistin resistance gene *mcr*). Tested isolates included five *S. aureus*, six coagulase-negative *Staphylococci* and six *Enterococci* among Gram positives, and seven *P. aeruginosa*, three *Acinetobacter baumannii*, eight *E. coli*, one *Enterobacter cloacae*, and five *K. pneumoniae* among Gram negatives (Table 1). The peptide showed strong activity against Gram-negative and Gram-positive bacteria with MICs ranging from 0.7 to 6.0 µM, and slightly better activity against the Gram positives. This finding confirmed data already available from different strains [26].

### 2.2. Frequency of Selection of Resistant Mutants after Exposure to SET-M33D

Selection of SET-M33D and colistin resistant mutants was performed on three reference strains (i.e., *E. coli* ATCC 25922, *K. pneumoniae* ATCC 13,833 for both agents and *S. aureus* ATCC 29,213 for SET-M33D only). SET-M33D MIC values were determined and resulted to be 1.5 µM for the selected *E. coli* and *S. aureus* strains and 3 µM for the *K. pneumoniae* strain, being overall consistent with what previously observed [26].

Colistin-resistant mutants were selected at a frequency of approximately 3 × 10^−8^ CFU for both the *E. coli* and *K. pneumoniae* strains. When selection using SET-M33D containing medium was attempted, a frequency of selection of approximately 6 × 10^−10^ CFU or lower was observed for both strains, suggesting an overall lower propensity of SET-M33D to select resistance with respect to colistin (Table 2).

Regarding the *S. aureus* reference strain, SET-M33D-resistant mutants were selected at a frequency 10 times higher than the tested Gram-negative strains (Table 2). In this case, no comparison of selection frequency with colistin-resistant mutants could be performed, due to the intrinsic resistance to colistin of this species.

### 2.3. In Vivo Antimicrobial Efficacy of SET-M33D

**Figure 2:**
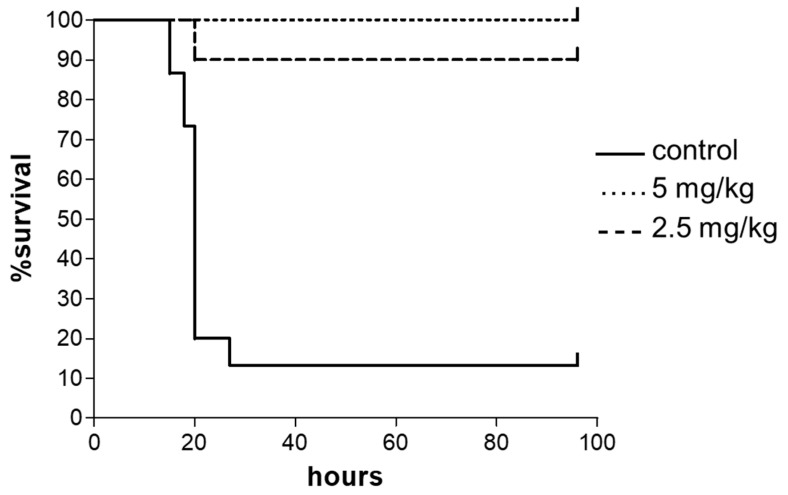
In vivo antibacterial activity of SET-M33D peptide. A total of 10 BALB-c mice/group (20 g) were injected i.p. with a lethal amount of *S. aureus* USA 300 (1 × 10^6^ CFU/mouse) and treated with SET-M33D 30 min, 3 and 6 h post-infection. Continuous line (control), injection with bacteria and treatment only with vehicle; dashed line, injection with bacteria and treatment with three injections of SET-M33D 5 mg/kg; dotted line, injection with bacteria and treatment with three injections of SET-M33D 2.5 mg/kg. *** *p* < 0.0001 Log-rank (Mantel-Cox) test; n = 10.

Antimicrobial activity of SET-M33D was evaluated in vivo in an animal model of infection with the highly virulent methicillin-resistant *S. aureus* (MRSA) strain USA 300, a lineage that has become a dominant cause of community associated MRSA infections in North America (Figure 2) [27,28]. Mice were infected i.p. with the smallest number of bacteria causing lethal infection, and treated i.p. with the peptides 30 min, 3 and 6 h post-infection. A 100% survival after 4 days was obtained with mice treated with 5 mg/kg of SET-M33D, while mice treated with 2.5 mg/kg of SET-M33D showed a mortality of 10%, confirming the potent in vivo activity of SET-M33D.

### 2.4. LPS and LTA Neutralization

**Figure 3:**
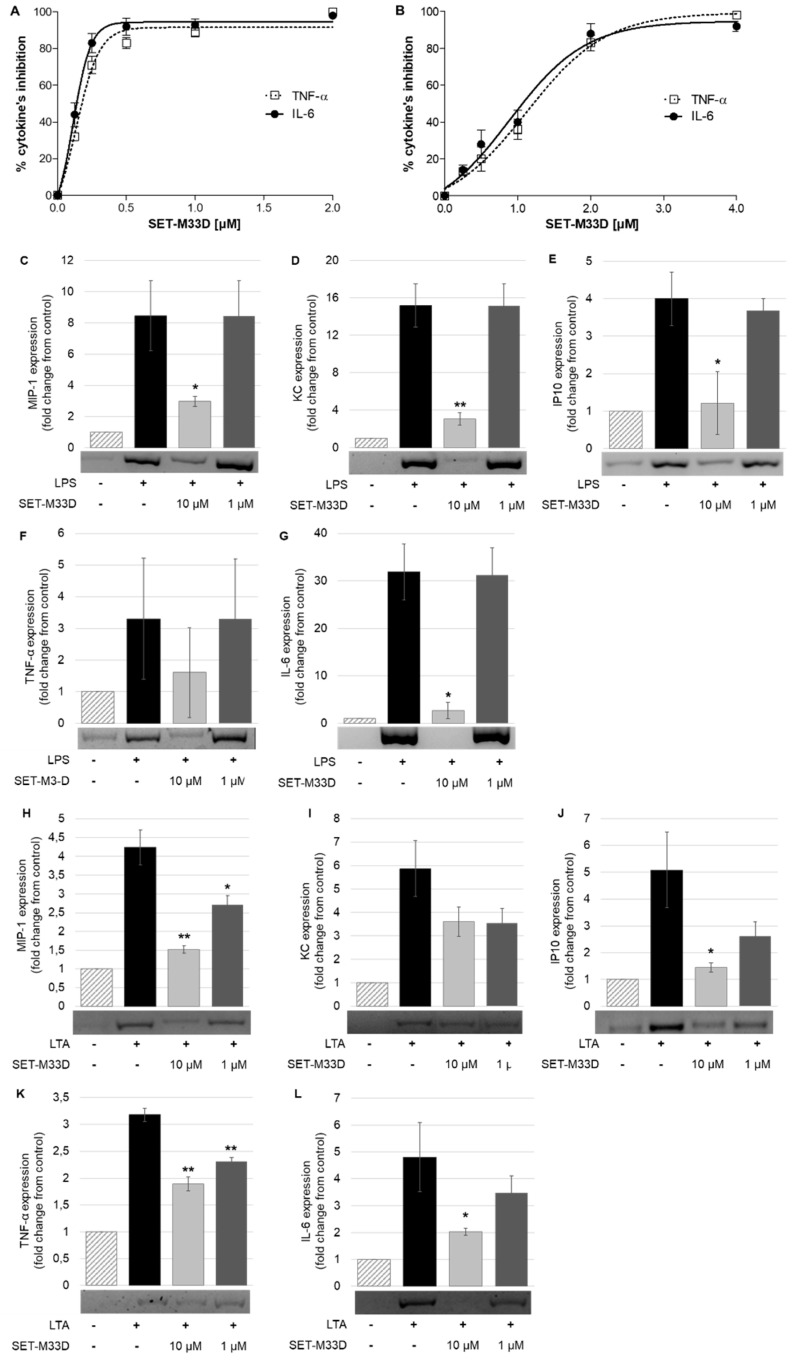
Effect of SET-M33D on protein release or gene expression of proinflammatory cytokines. (**A**,**B**) ELISA measurement of TNF-α and IL-6 produced by RAW264.7 cells after stimulation with LPS from *P. aeruginosa* (20 ng/mL) or LTA from *S. aureus* (2 µg/mL) in the presence of different concentration of SET-M33D. Data is expressed as percentage inhibition of cytokines with respect to LPS or LTA values (100%). Values are the mean ± SD of five independent experiments (n = 5). IC50s are reported in the text. (**C**–**L**) Gene expression of proinflammatory cytokines MIP1, KC, IP10, TNF-α, and IL-6 was analyzed by RT-PCR. RAW264.7 cells were stimulated with LPS from *E. coli* (**C**–**G**) or with LTA from *S. aureus* (**H**–**L**) in presence of SET-M33D at 10 or 1 µM. Densitometric analysis of cDNA bands (pictures under the columns) was carried out using ImageJ software. The reduction of cDNA is indicated as fold change with respect to control ± SD of two independent experiments (n = 2). * *p* < 0.05, ** *p* < 0.01 calculated using Student’s *t*-test with GraphPad Prism.

The ability of SET-M33D to neutralize LPS from *P. aeruginosa* and LTA from *S. aureus* was evaluated in terms of inhibition of protein production of the proinflammatory cytokines TNF-α and IL-6. RAW 264.7 macrophages were stimulated with 20 ng/mL of LPS for 4 h (Figure 3A) or with 2 µg/mL of LTA for 24 h (Figure 3B) and treated with different doses of SET-M33D. Then, the release of TNF-α and IL-6 in the medium was measured by ELISA. SET-M33D inhibited TNF-α and IL-6 with an IC50 of 1.36 and 1.30 µM, respectively, under LPS stimulation, and with an IC50 of 3.64 and 2.78 µM for TNF-α and IL-6, respectively, under LTA stimulation.

SET-M33D was also analyzed for its capacity to inhibit the gene expression of proinflammatory cytokines MIP1, KC, IP10, TNF-α, and IL6 induced by LPS or LTA in macrophages (Figure 3C–L). Gene expression analysis evaluated by RT-PCR showed that stimulation of RAW264.7 with LPS from *E. coli* or with LTA from *S. aureus* induced an increase in gene expression of all cytokines tested. In cells stimulated with LPS and then treated with 10 µM of SET-M33D, expression of proinflammatory cytokines was strongly inhibited (Figure 3C–G). SET-M33D inhibited 92% of IL6, 80% of KC, 70% of IP10, 65% of MIP1, and 52% of TNF-α. No inhibition of proinflammatory cytokines was observed in cells stimulated with LPS and treated with 1 µM of SET-M33D. When cells were stimulated with LTA and then treated with the peptide 10 µM the cytokine inhibition resulted as follows: >65% for IP10 and MIP1; 58% for IL6; around 40% for TNF-α and KC (Figure 3H–L). SET-M33D 1 µM produced a lower cytokine inhibition than the peptide 10 µM, still reducing the maximum level provoked by toxin stimulation (dark grey columns Figure 3H–L).

### 2.5. Inhibitory Effects of SET-M33D on COX-2 and iNOS Expression and Nitric Oxide Production

**Figure 4:**
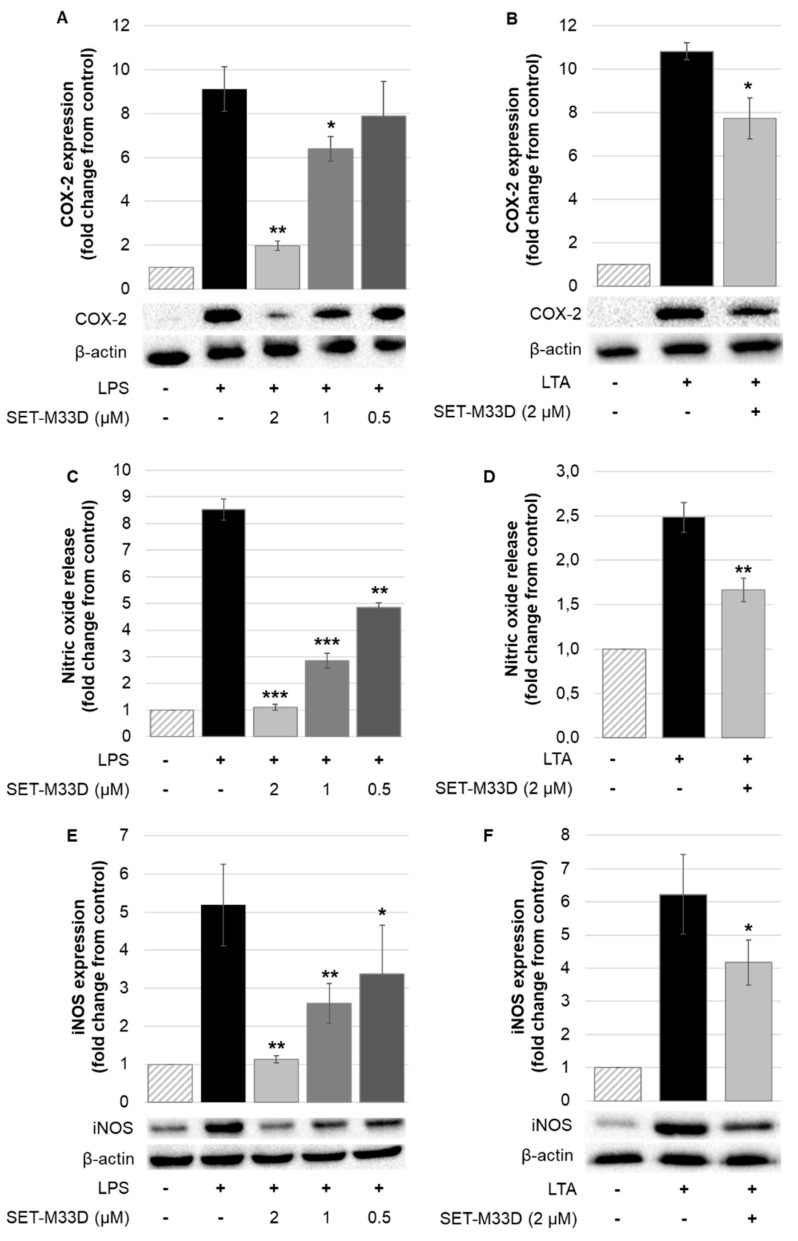
Effect of SET-M33D on COX-2 expression, nitric oxide release, and iNOS expression in mouse RAW 264.7 cells. Cells were stimulated with 20 ng/mL of LPS from *P. aeruginosa* for 4 h (**A**) or with 1 µg/mL for 24 h (**C**,**E**) or with LTA from *S. aureus* (2 µg/mL) for 24 h and treated with SET-M33D at the concentrations indicated. In some cases, (panels **B**,**D**,**F**) the lowest concentration of peptide did not produce any effect on the cytokine expression, so it was not reported in the figure. Relative COX-2 (**A**,**B**) and iNOS (**E**,**F**) expression levels were quantified by densitometry and normalized to β-actin protein. Values were determined as ratios between the intensities of protein bands in treated and untreated cells, assigning the value 1 to the control. The data is expressed as mean ± SD of three independent experiments (n = 3). Nitric oxide release (**C**,**D**) was measured as described in Section 4. The data is expressed as mean ± SD of four independent experiments (n = 4); * *p* < 0.05, ** *p* < 0.01 and *** *p* < 0.001 calculated using Student’s *t*-test and compared to the LPS group.

The inflammatory response is accompanied by an increase in several inflammatory mediators in addition to cytokines, such as PGE2 and nitric oxide, which are produced by COX-2 and iNOS enzymes. To investigate the possible involvement of COX-2, RAW264.7 cells were treated with LPS from *P. aeruginosa* (20 ng/mL) alone or in combination with SET-M33D at the concentrations indicated in Figure 4A and COX-2 expression was evaluated by Western blot. When the cells were exposed to LPS alone, a sharp increase in COX-2 expression was observed, while after cell treatment with different doses of the peptide, COX-2 expression dropped in a dose-dependent manner, reaching the minimum at SET-M33D 2 µM (a reduction of 78.4% ± 1.15) (Figure 4A). Cells were also treated with LTA from *S. aureus* (2 µg/mL) alone and with SET-M33D (2 µM) for 24 h. LTA induced a considerable increase in COX-2 expression, and the peptide downregulated its expression by 30.1% (±8.9) (Figure 4B).

The activation of COX-2 protein is closely related to induction of nitric oxide synthase (iNOS) and the resulting production of nitric oxide. RAW 264.7 cells were stimulated with LPS (1 µg/mL) alone and with SET-M33D (0.5–2 µM) for 24 h, and nitric oxide production was measured as described in Section 4. LPS caused a significant release of nitric oxide (8.5-fold over unstimulated cells) which was dose-dependently attenuated by SET-M33D with maximal effect (87.0% ± 1.8 inhibition) at 2 µM peptide (Figure 4C). Inhibition of nitric oxide release by SET-M33D under LTA (2 µg/mL) stimulation for 24 h was 32.2% (± 6.2) using the concentrated peptide 2 µM (Figure 4D) (LTA alone produced a 2.8-fold increase over unstimulated cells).

To examine whether inhibition by SET-M33D could be attributed to its modulation of iNOS protein expression, Western blot analysis was carried out. A certain amount of iNOS protein was produced under LPS and LTA stimulation and it was reduced by SET-M33D in a concentration-dependent manner. SET-M33D (2 µM) reduced iNOS expression, restoring expression to basal level when cells were stimulated with LPS (Figure 4E), and inhibiting protein expression by about 32.6% (± 3.7) when cells were stimulated with LTA (Figure 4F).

### 2.6. Effect of SET-M33D on NF-κB Nuclear Translocation

**Figure 5:**
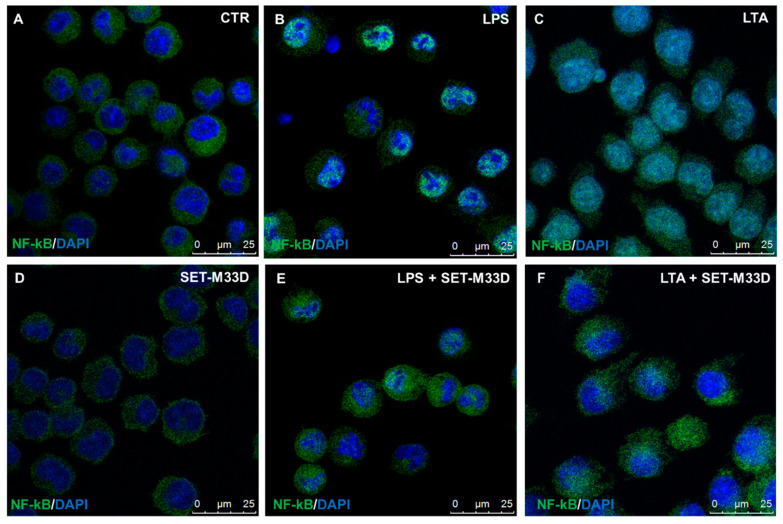
Immunofluorescence staining showing NF-kB/p65 distribution (green signal) in healthy RAW264.7 cells (**A**), stimulated with LPS from *P. aeruginosa* (**B**) or LTA from *S. aureus* (**C**) for 4 h, or treated with SET-M33D 2 µM for 24 h, alone (**D**), with LPS and SET-M33D (**E**), or with LTA and SET-M33D (**F**). Nuclei were stained with DAPI (blue). Translocation of NF-kB was analyzed by confocal microscopy. The shift of green signal from the cytoplasm to the nucleus is shown by merged colors in the nucleus in LPS- and LTA-stimulated cells (**B**,**C**). In the presence of SET-M33D, colocalization of the signal in the nucleus is abolished (**E**,**F**). The peptide alone does not produce any merging of colors.

NF-kB/p65, a member of the NF-kB protein family, is transferred to the nucleus in response to stimulation with LPS or LTA. NF-kB/p65 translocations into macrophages were analyzed by immunofluorescence after LPS or LTA stimulation and SET-M33D treatment. NF-kB/p65 was located in the cytoplasm in the control group (Figure 5A, sharp green signal), while in LPS- or LTA-stimulated cells, it translocated to the nucleus (Figure 5B,C), as shown by merging of colors. Treatment with SET-M33D clearly restored the NF-kB/p65 to the cytoplasm (Figure 5E,F). SET-M33D alone did not produce any change in NF-kB/p65 translocation (Figure 5D).

### 2.7. Cytotoxicity In Vitro

**Figure 6:**
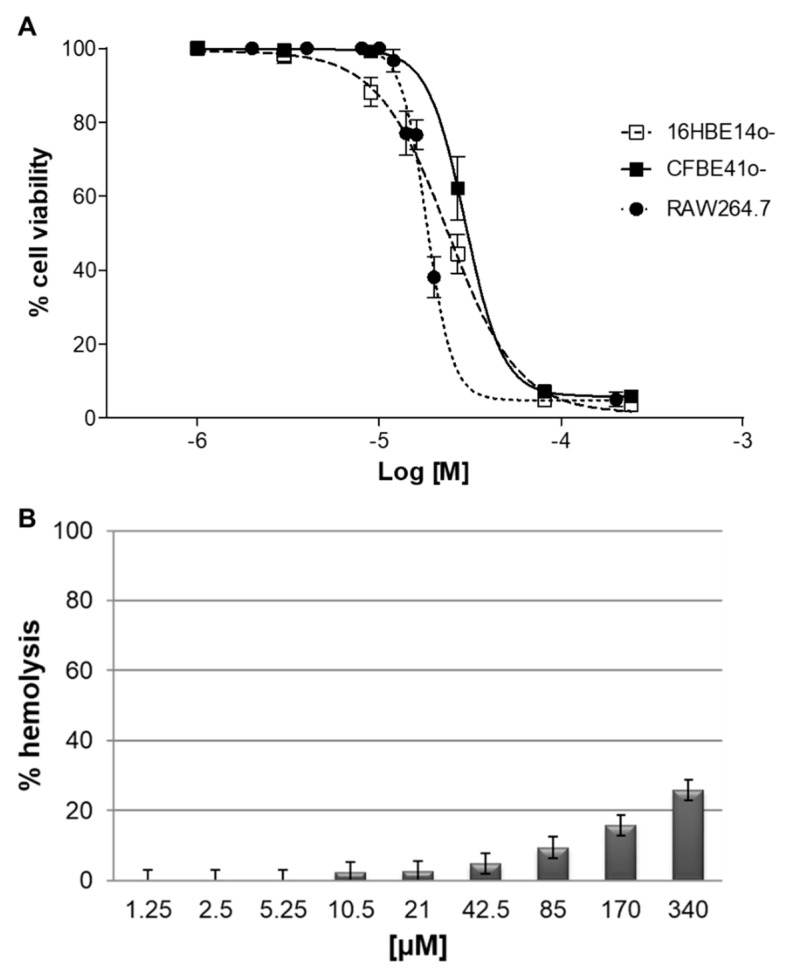
Cytotoxic and hemolytic effect of SET-M33D in vitro. RAW 264.7, 16HBE14o^-^, and CFBE41o^-^ cells (**A**) were incubated with increasing concentrations of SET-M33D for 48 h. Cell viability is expressed as a percentage with respect to untreated cells ± SD. The experiment was performed twice in triplicate; n = 6. (**B**) Hemolytic activity of SET-M33D is reported as a percentage ± SD of red blood cell hemolysis after incubation for 24 h at 37 °C.

The cytotoxic effect of SET-M33D was analyzed in vitro in human (16HBE14o^-^ and CFBE41o^-^) and mouse cells (RAW 264.7 macrophages) (Figure 6A), which were exposed to increasing concentrations of peptide, from 1 to 100 µM for 48 h. The IC50 of SET-M33D proved to be 2.4 × 10^−5^ M, 2.9 × 10^−5^ M, and 1.8 × 10^−5^ M in 16HBE14o^-^, CFBE41o^-^, and RAW 264.7, respectively.

SET-M33D was also analyzed for its capacity to damage red blood cells (Figure 6B). The peptide did not cause more than 25% hemolysis, even at a concentration of 340 µM, which is more than 55 times the highest MIC reported in Table 1.

### 2.8. Acute Toxicity In Vivo

**Figure 7:**
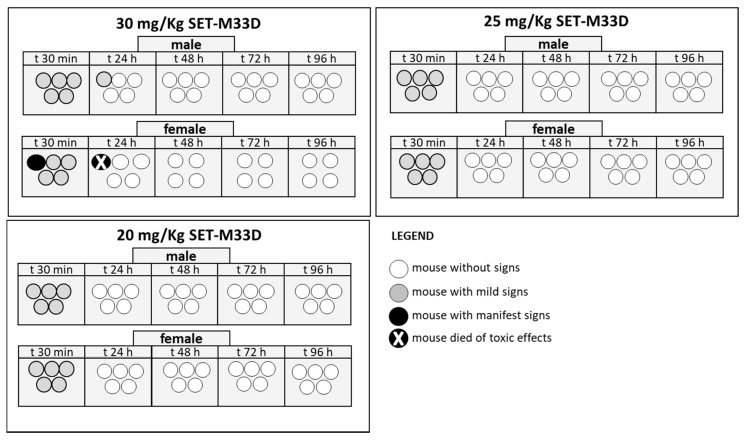
Acute in vivo toxicity of SET-M33D at 30, 25, and 20 mg/kg, given in a single dose. Ten mice/group (each circle represents a mouse), five males and five females, were inoculated i.v. with SET-M33D and were monitored for 96 h. Different scales of grey indicate severity of signs as described in the legend.

CD-1 mice were treated i.v. with SET-M33D 30, 25, or 20 mg/kg in a single dose (Figure 7), and were monitored for 4 days. No signs of toxicity were observed at 20 and 25 mg/kg in any animal. At 30 mg/kg, SET-M33D caused 10% mortality after 24 h. All the mild signs of toxicity recorded immediately after inoculation disappeared in live animals within 24 h. No significant variation in body weight was detected (not shown).

## 3. Discussion

Antimicrobial peptides cannot be considered a complete alternative to traditional antibiotics, because with respect to the latter, they generally have lower activity, poor stability, and sometimes production difficulties. However, they can play a very important role in the difficult fight against bacteria because they are often active against bacteria resistant to traditional antibiotics, and may not lead to selection of resistant bacteria [29,30]. Furthermore, some have a multifactorial mechanism of action: they kill bacteria while neutralizing bacterial toxins, thus strongly reducing the inflammatory process triggered by living or dead bacteria [31,32].

The peptide SET-M33D is a synthetic tetrameric molecule synthesized with D amino acids. It is derived from a previous peptide synthesized with L amino acids [20,22], which only showed activity against Gram-negative bacteria. SET-M33D also proved to be active against Gram-positive pathogens, by virtue of its stability to the proteases of these bacteria [26]. Here we described its strong activity against a panel of Gram-positive and Gram-negative bacteria, including clinical isolates with multidrug-resistant phenotypes, as well as its low ability to cause selection of resistant mutants, its lack of hemolytic action, and its anti-inflammatory activity due to neutralization of LPS and LTA derived from pathogens of clinical interest. We demonstrated that the neutralization of bacterial toxins provoked a strong decrease in cytokines TNF-α, IL-6, MIP-1, KC, IP10, and in enzymes iNOS and COX-2, which are considered crucial agents for triggering and fostering inflammatory processes [33]. Its anti-inflammatory property linked to LPS and LTA neutralization was further confirmed by inhibition of NF-kB translocation into the cell nucleus. NF-kB activation and translocation is due to a series of cell events related to pathogen-associated molecular patterns, and LTA and primarily LPS have long been considered to be the prototypical class of such patterns [34,35].

By virtue of its dual activity, namely killing bacteria and restraining inflammation, this molecule could be very suitable for treating lung infections in cystic fibrosis patients, where bacterial growth and uncontrolled inflammation together play a crucial role in progression of lung damage and evolution of the disease. It is noteworthy that *S. aureus* and *P. aeruginosa*, the main pathogens involved in lung infections in cystic fibrosis patients [36], are among the most susceptible species to SET-M33D.

Furthermore, the promising in vivo efficacy and low in vivo toxicity reported here allow us to predict a favorable therapeutic index for eventual clinical use of this peptide.

## 4. Materials and Methods

### 4.1. Materials

Dulbecco’s modified Eagle’s medium (DMEM), fetal bovine serum (FBS), and fetal calf serum (FCS) were from Euroclone (Pero, Mi, Italy). LTA-SA purified from *S. aureus* was purchased from InvivoGen (CA, USA); LPS from *P. aeruginosa* (serotype 10, strain ATCC27316, L 9143), LPS from *E. Coli* (026:B6, L 8274), and LTA from *S. aureus* (L 2515) were purchased from Sigma Aldrich. ELISA kits for IL-6 and TNF-α determinations were supplied from BioLegend (CA, USA) and PeproTech, (Rocky Hill, NJ), respectively. Horseradish peroxidase-conjugated anti-rabbit and anti-mouse IgG and anti-COX-2 antibodies were from Thermo Fisher (Rockford, IL, USA); anti-iNOS and anti-NF-kB p65 antibodies were purchased from Cell Signaling Technology (Danvers, MA, USA); anti-β-actin was purchased from Santa Cruz Biotechnology, Inc (CA, USA). Alexa Fluor 488-conjugated secondary antibodies were from Invitrogen (Molecular Probes, Invitrogen, Carlsbad, CA, USA).

PVDF membranes were from Bio-Rad (Richmond, USA); the reagents for enhanced chemiluminescence (ECL) detection were obtained from Amersham Life Science (Pittsburgh, PA, USA). Sigma Aldrich (St. Louis, Mo, USA) provided [3-(4,5-dimethylthiazol-2-yl)-2,5-diphenyltetrazolium bromide] (MTT), Griess reagent, protease inhibitor cocktail, and other reagents.

### 4.2. Peptide Synthesis

The peptide SET-M33D, (kkirvrlsa)_4_K_2_KβA-OH was synthesized on solid phase using a multiple peptide synthesizer, Syro (MultiSynTech, Witten, Germany), by standard Fmoc chemistry. The resin used was a Wang type resin, TentaGel-PHB 4 branch βAla (Rapp Polymere, Germany), which carries the branching core in L-form, Fmoc_4_-Lys_2_-Lys-β-Ala, as previously described [26]. Side chain protecting groups were 2,2,4,6,7-pentamethyldihydrobenzofuran-5-sulfonyl for R, t-butoxycarbonyl for K and t-butyl for S. SET-M33D was synthesized using Fmoc-D-amino acids. The final product was cleaved from the solid support, deprotected by treatment with TFA containing triisopropylsilane and water (95/2.5/2.5) and precipitated with diethyl ether. Crude peptide was purified by reversed-phase chromatography on a Phenomenex Jupiter C18 column (300 Å, 10 mm, 250,610 mm), using 0.1% TFA/water as eluent A and methanol as eluent B, in a linear gradient from 80% A to 20% A in 30 min. The purified peptide was obtained as trifluoroacetate salts (TFacetate). The exchange from TFacetate to acetate form was carried out using a quaternary ammonium resin in acetate form (AG1-X8, 100–200 mesh, 1.2 meq/mL capacity, Bio-Rad). The resin-to-peptide ratio was 2000:1, resin and peptide were stirred for 1 h, the resin was filtered off, washed extensively, and the peptide recovered and freeze-dried. Final peptide purity and identity were confirmed by reversed phase chromatography on a Phenomenex Jupiter C18 analytical column (300 Å, 5 mm, 25,064.6 mm), (kkirvrlsa)_4_K_2_KβA-NH_2_ RT = 18 min and by mass spectrometry with a Bruker Daltonics ultraflex MALDI TOF/TOF, M^+^(found) = 4682.86.

### 4.3. Susceptibility Testing

SET-M33D minimum inhibitory concentrations (MICs) were determined in triplicate on a panel of reference and clinical strains using a reference microdilution assay, performed according to the guidelines of the Clinical and Laboratory Standards Institute (CLSI, Methods for Dilution Antimicrobial Susceptibility Tests for Bacteria That Grow Aerobically, M07, 11th ed) as previously described [26]. Briefly, strains were grown on Mueller–Hinton agar (MHA) plates and a single colony for each strain was picked using a sterile cotton swab, streaked in sterile cation-supplemented Mueller–Hinton broth (MHB) (Becton Dickinson, Franklin Lakes, NJ, USA) and measured with a densitometer (Densicheck, bioMèrieux, Marcy l’Etoile, France) up to a 0.5 McFarland density. A total of 50 µL of each bacterial suspension were used to inoculate wells of a microtiter plate containing an equal volume of serial doubling dilutions of SET-M33D performed in the same suspension media used for bacterial inocula. Assays were performed using a final bacterial inoculum of 5 × 10^4^ CFU/well in a volume of 100 µL. MIC values were recorded after plates incubation at 35 °C for 18–20 h. Assays were performed in triplicate and the median MIC values were reported.

### 4.4. Selection of Resistant Mutants

Selection of resistant mutants was carried out on reference strains using an MHB-based selection medium containing 1% low electro-osmosis agarose as solidifying agent and 12 µM SET-M33D. Strains were grown on MHA plates and a single colony for each strain was picked using a sterile cotton swab, streaked in sterile cation-supplemented MHB and grown at 37 °C to OD_600_ 0.5. Up to 3 × 10^9^ colony forming units (CFU) were spread on Petri dishes containing 15 mL of the selection medium. The same selection medium containing colistin in equimolar concentration with respect to SET-M33D was used as control for the selection of colistin-resistant mutants of the *E. coli* and *K. pneumoniae* strains. Plates were incubated for 16–18 h at 37 °C and colonies grown on the SET-M33D and colistin selection media were counted. Viable cell counts of the used bacterial suspension were obtained by plating appropriate serial dilution on nonselective MHA plates. Three replicates using distinct cultures of each strain were performed. The mutation frequency was calculated as the number of mutants divided by the viable cell count.

### 4.5. In Vivo Efficacy

Balb-c mice (20 g) were infected i.p. with a lethal amount of methicillin-resistant *S. aureus* (MRSA) strains USA 300 (1 × 10^6^ CFU/mouse in 500 µL PBS with 7% mucin; mucin from porcine stomach, type II, Sigma-Aldrich). The mice were treated three times with i.p. injection of SET-M33D, diluted in 0.9% NaCl solution, at 5 and 2.5 mg/kg, 0, 3, and 6 h post-infection. Control animals received only vehicle (PBS). Groups consisted of 10 animals each. Moribund animals were killed humanely to avoid unnecessary distress.

### 4.6. Cell Culture

Murine macrophage cell line RAW 264.7 was from ECACC (European Collection of Cell Cultures, Salisbury, UK) and was maintained in DMEM supplemented with 10% FBS, antibiotics (100 U/mL penicillin G, 100 μg/mL streptomycin) and L-glutamine (2 mM) at 37 °C in a humidified incubator under a 5% CO_2_ atmosphere. 16HBE14o^-^ (human bronchial epithelial cells) and CFBE41o^-^ (cystic fibrosis bronchial epithelial cells with DF508 mutation in the CFTR gene) were provided by Dr. Dieter Gruenert (California Pacific Medical Center Research Institute, San Francisco, CA, USA) and maintained in Eagle’s minimum essential medium (EMEM) supplemented with 10% FCS, 0.1 mM nonessential amino acids (NEAA), 2 mM L-glutamine, 100 μg/mL streptomycin, and 100 U/mL penicillin G, at 37 °C in a 5% CO_2_ incubator.

### 4.7. TNF-α and IL-6 Quantification

RAW 264.7 cells were seeded in 24-well plates (2 × 10^5^ cells/well) and incubated overnight at 37 °C. They were then treated with (i) LPS from *P. aeruginosa* 20 ng/mL alone or together with SET-M33D (0.12–2 µM) for 4 h; (ii) LTA from *S. aureus* (2 µg/mL) alone or together with SET-M33D (0.25–4 µM) for 24 h. TNF-α and IL-6 levels were determined in the medium using commercially available ELISA kits: Peprotech (Rocky Hill, NJ, USA) for TNF-α and Biolegend (San Diego, CA, USA) for IL-6. Cytokine levels in cells treated only with LPS and LTA, were taken as 100 and the data was expressed as mean percentage inhibition. IC50 values were calculated by GraphPad Prism version 5.03 for Windows.

### 4.8. Gene Expression of Proinflammatory Factors

RAW264.7 cells were seeded in six-well plates (5 × 10^5^ cells per well) with complete medium and cultured in a CO_2_ incubator overnight. They were stimulated with 20 ng/mL LPS from *E. coli* or 1 µg/mL LTA from *S. aureus* in the presence of 10 or 1 µM peptide SET-M33D in DMEM for 6 h. Total RNA was extracted using a NucleoSpin RNA kit (Macherey-Nagel) according to the manufacturer’s instructions. RNA was quantified by spectrophotometry at 260 nm and its quality assessed by 260/280 nm ratio. Then, 500 ng of RNA was used for each RT-PCR reaction. One step RT-PCR (QIAGEN) was used for retrotranscription and mouse cDNA amplification of MIP-1α (368 bp), TNF-α (795 bp), IL-6 (474 bp), KC (391 bp), and IP10 (127 bp). The following oligonucleotides were used as primers: MIP-1 primers were 5′-ATG AAG CTC TGC GTG TCT GC-3′ (sense), 5′-TGA GGA GCA AGG ACG CTT CT-3′ (antisense); TNF-α primers were 5′-GTT CTG TCC CTT TCA CTC ACT G-3′ (sense), 5′-GGT AGA GAA TGG ATG AAC ACC-3′ (antisense); IL-6 primers were 5′-CAT GTT CTC TGG GAA ATC GTG G-3′ (sense), 5′-AAC GCA CTA GGT TTG CCGA GTA-3′ (antisense); KC primers were 5′-ACT GCA CCC AAA CCG AAG TCA TAG-3′ (sense), 5′-GCA CAG TGG TTG ACA CTT AGT GGT-3′ (antisense); IP-10 primers were 5′-GCC GTC ATT TTC TGC CTC AT-3′ (sense), 5′-GCT TCC CTA TGG CCC TCA TT-3′ (antisense).

The following PCR conditions were applied: for TNF-α, 25 denaturing cycles at 94 °C for 60 s, annealing at 55 °C for 90 s and extension at 72 °C for 60 s; for MIP1 20 denaturing cycles at 94 °C for 60 s, annealing at 55 °C for 90 s and extension at 72 °C for 60 s; for KC 30 denaturing cycles at 94 °C for 60 s, annealing at 54 °C (KC) and 55 °C (MCP1) for 60 s and extension at 72 °C for 60 s: for IP10 25 denaturing cycles at 94 °C for 60 s, annealing at 54 °C for 60 s and extension at 72 °C for 60 s; for IL-6 30 denaturing cycles at 94 °C for 30 s, annealing at 57 °C for 30 s and extension at 72 °C for 60 s.

### 4.9. Nitrite Assay

Nitric oxide levels in supernatants were measured by determination of total nitrite from the Griess reaction. RAW 264.7 cells were seeded at a cell density of 1 × 10^5^ cells/well in 96-well plates and incubated overnight at 37 °C. Cells were treated for 24 h with LPS 1 µg/mL alone or plus SET-M33D from 0.5 to 2 µM; LTA 2 µg/mL alone or with SET-M33D 2 µM. Culture supernatants were mixed with equal volume of Griess reagent at room temperature for 20 min in the dark; absorbance at 540 nm was measured and nitrite concentration (NO_2_^−^) was determined using sodium nitrite (NaNO_2_) as standard.

### 4.10. Western Immunoblot Analysis

RAW 264.7 were treated with (i) LPS from *P. aeruginosa* (20 ng/mL or 1 µg/mL) alone or with SET-M33D (0.5, 1, and 2 µM) for 4 or 24 h; (ii) LTA from *S. aureus* (2 µg/mL) alone or with SET-M33D (2 µM) for 24 h. After treatment, the cells were solubilized in lysis buffer (PBS 10 mM, 1% Nonidet P40, 0.5% sodium deoxycholate, 0.1% SDS, Protease Inhibitor Cocktail, 1 mM Na_3_VO_4_, 5 mM DTT) for 30 min on ice and then centrifuged at 14,000× *g* for 10 min. The supernatants were collected, and the proteins were separated on SDS-PAGE and transferred to PVDF membrane. After blocking with TBST (100 mM NaCl, 10 mM Tris-HCl pH 7.5, 0.1% Tween 20) containing 5% non-fat dry milk overnight at 4 °C, the membranes were incubated with primary anti-COX-2, anti-iNOS, or anti-β-actin antibody for 1 h at room temperature. After washing with TBST, the membranes were incubated with HRP-conjugated secondary antibodies for 1 h. Blots were developed by the ECL system. Densitometry analysis of bands was done using the ImageJ gel system.

### 4.11. Immunofluorescence

RAW264.7 cells were plated at a density of 5 × 10^4^ cells/well in 24-well plates with cover glass slides and stimulated with 20 ng/mL LPS from *P. aeruginosa* alone or in the presence of 2 and 10 μM SET-M33D for 4 h or 2 µg/mL LTA from *S. aureus* with or without 2 µM SET-M33D for 24 h at 37 °C. After washing with PBS, cells were fixed in 4% paraformaldehyde for 15 min at room temperature and permeabilized with PBS containing 0.3% TritonX-100 and 5% normal bovine serum for 1 h. Cells were incubated with rabbit anti-NF-κB p65 mAb diluted 1:250 in PBS with 1% BSA and 0.3% Triton X-100 overnight at 4 °C. After washing with PBS, antibody-bound cells were incubated with Alexa Fluor 488 conjugated anti-rabbit IgG secondary antibodies (1:2000) for 30 min at room temperature. Cell nuclei were counterstained with DAPI (0.5 µg/mL) for 10 min at room temperature. Coverslips were mounted on slides and examined with a Leica TCS SP5 confocal microscope (Leica Microsystems, Mannheim, Germany).

### 4.12. Cell Viability Assay

16HBE14o^-^ and CFBE41o^-^ were plated at a density of 2.5 × 10^4^ per well in 96-well microplates, previously incubated with coating solution (88% LHC basal medium, 10% bovine serum albumin, 30 g/mL bovine collagen type I and 1% human fibronectin), while RAW 264.7 cells were seeded in 96-well plates (5 × 10^3^ per well) and incubated for 24 h at 37 °C in a 5% CO_2_ atmosphere. Cells were treated with 100 µL fresh medium containing SET-M33D at different concentrations for 48 h. Then 20 µl MTT (5 mg/mL) was added to each well and the plate was incubated at 37 °C for 3 h. Finally, 120 µL HCl 4 mM in isopropanol was added to each well to solubilize the formazan crystals. Optical density was measured with a microplate reader (Bio-Rad, Hercules, CA, USA) at 570 nm. Cell viability was calculated comparing the values of treated groups with those of untreated cells. IC50 were calculated using GraphPad Prism 5.03 software.

### 4.13. Hemolytic Activity

Whole human blood in EDTA was centrifuged (1100 g) for 10 min. Red blood cells diluted 1:100 in physiological solution (0.9% NaCl) were incubated for 24 h at 37 °C with serial dilution of SET-M33D from 1.25 to 340 µM. The absorbance of the supernatants was determined in a 96-well plate at 490 nm with a micro plate reader. Data for 100% hemolysis was obtained by adding 0.1% TritonX-100 in water to cells. The negative control was physiological solution. The hemolysis rates of the peptides were calculated with the following equation: (%) = (A peptide—A physiological solution)/(A triton—A physiological solution) × 100%; where A = absorbance.

### 4.14. Acute Toxicity In Vivo

Animal procedures were approved by the Italian Ministry of Health, 14th January 2016, protocol 34/2016-PR. Eight-week-old BALB/c female mice (Charles River) were used in all experiments. The animals were maintained and handled in accordance with the Guidelines for Accommodation and Care of Animals (European Convention for the Protection of Vertebrate Animals Used for Experimental and Other Scientific Purposes) and internal guidelines.

CD-1 mice were treated by a single i.v. administration of different amounts (30, 25, and 20 mg/kg) of SET-M33D, diluted in physiological solution (0.9% NaCl). Groups consisted of 10 animals, five females and five males each. Signs of toxicity were monitored four times a day by visual inspection. A toxicity score was assigned for the following signs: wiry coat and poor motility = mild signs; very wiry coat, abundant lachrymation and poor motility even under stimulation = manifest signs. Animals were observed for 4 days after inoculation of the peptide. Mice were weighed every day from arrival to the last day of the experiment. Moribund animals were killed humanely to avoid unnecessary distress.

### 4.15. Statistical Analysis

Quantitative data was expressed as the mean ± standard deviation (S.D.) of three to five separate experiments. Statistical analysis was performed using Student’s one-tailed *t*-test. Probability values of *p* < 0.05 were considered statistically significant.

## 5. Conclusions

This study is a further development of SET-M33D as an active drug in eradicating Gram-negative and Gram-positive bacteria. Earlier studies showed that SET-M33D could eradicate biofilms of Gram-negative and Gram-positive bacteria and that the all-D configuration was the key for the wider spectrum of activity compared to the parent compound SET-M33L which was only active against Gram-negative [26].

The next step of development, before filing for starting clinical trials, will be the preclinical characterization in animals aimed at the evaluation of safety pharmacology and dose range finding. Chemistry, Manufacturing, and Control evaluations on SET-M33D production are ongoing.

All these features make the peptide SET-M33D a strong candidate for the full drug development phase of a new broad spectrum antibacterial agent.

## Figures and Tables

**Table 1:** MIC values of SET-M33D peptide on selected strains of major pathogen species.

Strain	Species	Relevant Features ^a^	Relevant Resistance Genotype	MIC (µM)
ATCC 700,699 Mu50	*Staphylococcus aureus*	Reference strain, MR, GLY		1.5
USA 300	*Staphylococcus aureus*	MR		1.5
SI-B	*Staphylococcus aureus*	MR		3
SI-R	*Staphylococcus aureus*	MR		1.5
FI-F	*Staphylococcus aureus*	none		1.5
FI-4LNZ	*Staphylococcus epidermidis*	LIN	*cfr*	0.7
4761/1	*Staphylococcus epidermidis*	None		0.3
ATCC 27840	*Staphylococcus capitis*	Reference strain		0.3
FI-1	*Staphylococcus saprophyticus*	None		0.7
FI-2	*Staphylococcus saprophyticus*	None		1.5
FI-3	*Staphylococcus saprophyticus*	MR		1.5
*ATCC 29212*	*Enterococcus faecalis*	Reference strain		3
FI-4	*Enterococcus faecalis*	FQ, GLY		1.5
FI-5	*Enterococcus faecalis*	FQ		3
FI-6	*Enterococcus faecalis*	FQ		3
FI81B1	*Enterococcus faecium*	GLY		0.7
FI81B2	*Enterococcus faecium*	None		0.7
PAO-1	*Pseudomonas aeruginosa*	Reference strain		0.7
FI-8	*Pseudomonas aeruginosa*	CARB, β/I, ESC, FQ, AG		3
FI-9	*Pseudomonas aeruginosa*	CARB, β/I, ESC, FQ, AG	*bla*_VIM_	3
FI-10	*Pseudomonas aeruginosa*	CARB, β/I, ESC, FQ, AG		3
FI-11	*Pseudomonas aeruginosa*	CARB, β/I, ESC, FQ, AG	*bla*_VIM_	3
VR-143/97	*Pseudomonas aeruginosa*	CARB, β/I, ESC, FQ, AG	*bla*_VIM-1_	3.0
OBG6-1	*Pseudomonas aeruginosa*	CARB, β/I, ESC, FQ, AG	*bla*_IMP-13_	1.5
RUH 875	*Acinetobacter baumannii*	Reference strain, European clone I		3.0
RUH 134	*Acinetobacter baumannii*	Reference strain, European clone II		3.0
MR 157	*Acinetobacter baumannii*	CARB, β/I, ESC, FQ, AG	*bla*_OXA-58_	6.0
FI-13	*Escherichia coli*	CARB, ESC, AG	*bla*_KPC_	6
FI-14	*Escherichia coli*	ESC, FQ, AG		1.5
FI-19	*Escherichia coli*	AG, COL	*mcr1.5*	6
FI-21	*Escherichia coli*	COL	*mcr1*	6
FI-22	*Escherichia coli*	COL	*mcr1*	3
FI-23	*Escherichia coli*	COL	*mcr1*	3
FI-24	*Escherichia coli*	COL	*mcr1*	3
MO-287	*Escherichia coli*	CARB, β/I, ESC, FQ, AG	*bla*_NDM-1_	3.0
W03AN0041	*Enterobacter cloacae*	ESC	*bla*_SHV-12_	1.5
FI-15	*Klebsiella pneumoniae*	CARB, β/I, ESC, FQ, AG	*bla*_KPC_	3
FI-16	*Klebsiella pneumoniae*	CARB, β/I, ESC, FQ, AG	*bla*_KPC_	3
FI-17	*Klebsiella pneumoniae*	CARB, β/I, ESC, FQ	*bla*_KPC_	6
FI-18	*Klebsiella pneumoniae*	CARB, β/I, ESC, FQ, AG	*bla*_KPC_	3
FI-20	*Klebsiella pneumoniae*	CARB, β/I, ESC, FQ, AG, COL	*mcr1.2*, *bla*_KPC_	6

^a^ MR, methicillin resistant; CARB, carbapenem resistant; β/I, β-lactam/inhibitor combination resistant; ESC, extended-spectrum cephalosporin resistant; FQ, fluoroquinolone resistant; AG, aminoglycoside resistant; COL, colistin resistant, GLY, glycopeptide resistant; LIN, linezolid resistant.

**Table 2:** Frequency of selection (mean ± SD) of resistant mutants on three reference strains after exposure to SET-M33D and to colistin.

Strain	MIC SET-M33D (µM)	MIC Colistin (µM)	Mutation Frequency on SET-M33D	Mutation Frequency on Colistin
*E. coli* ATCC 25922	1.5	0.35	<6.6 × 10^−10^ *	3.8 × 10^−8^ ± 1.9 × 10^−8^
*K. pneumoniae* ATCC 13833	3	1.5	<6.6 × 10^−10^ *	3.2 × 10^−8^ ± 3.5 × 10^−8^
*S. aureus* ATCC 29213	1.5	-	5.9 × 10 ^−9^ ± 2.8 × 10^−9^	-

* In these cases, the frequency of mutation was reported as lower than the observed median values since no mutants were selected in the experimental conditions.
